# Obstetrics

**Published:** 1893-04

**Authors:** 


### Book Reviews.

OBSIETRICS—By Charles W. Hayt, M. D., House Physician, Nursery and Children's Hospital, New York. Being volume 11 of THE STUDENT'S QUIZ SERIES. Pocket size, 190 pages, $1.00. Philadelphia, Lea Brothers & Co., 1892.

THIS is a neat little manual of 190 pages of concise and well written matter, and is evidently superior to the ordinary Quiz Series. It contains the most approved teachings and principles in the obstetrical science. It is not intended to take the place of the standard works on this subject, so it can be recommended to any one who wishes to review the subject in the scope ordinarially given in manuals. W. A. C.

